# Estimating Leaf Area Index (LAI) in Vineyards Using the PocketLAI Smart-App

**DOI:** 10.3390/s16122004

**Published:** 2016-11-26

**Authors:** Francesca Orlando, Ermes Movedi, Davide Coduto, Simone Parisi, Lucio Brancadoro, Valentina Pagani, Tommaso Guarneri, Roberto Confalonieri

**Affiliations:** 1 Department of Agricultural and Environmental Sciences—Production, Land, Agrienergy, Università degli Studi di Milano, via Celoria 2, I-20133 Milan, Italy; ermes.movedi@unimi.it (E.M.); davide.coduto@studenti.unimi.it (D.C.); meteoclima@hotmail.it (S.P.); lucio.brancadoro@unimi.it (L.B.); tommaso.guarneri@unimi.it (T.G.); 2 Cassandra Lab, Università degli Studi di Milano, via Celoria 2, I-20133 Milan, Italy; valentina.pagani@unimi.it (V.P.); roberto.confalonieri@unimi.it (R.C.); 3 Department of Economics, Management, and Quantitative Methods, Università degli Studi di Milano, via Celoria 2, I-20133 Milan, Italy

**Keywords:** hemispherical photography, leaf area index, plant vigour, smart-app, *Vitis vinifera*

## Abstract

Estimating leaf area index (LAI) of *Vitis vinifera* using indirect methods involves some critical issues, related to its discontinuous and non-homogeneous canopy. This study evaluates the smart app PocketLAI and hemispherical photography in vineyards against destructive LAI measurements. Data were collected during six surveys in an experimental site characterized by a high level of heterogeneity among plants, allowing us to explore a wide range of LAI values. During the last survey, the possibility to combine remote sensing data and in-situ PocketLAI estimates (smart scouting) was evaluated. Results showed a good agreement between PocketLAI data and direct measurements, especially for LAI ranging from 0.13 to 1.41 (*R*^2^ = 0.94, RRMSE = 17.27%), whereas the accuracy decreased when an outlying value (vineyard LAI = 2.84) was included (*R*^2^ = 0.77, RRMSE = 43.00%), due to the saturation effect in case of very dense canopies arising from lack of green pruning. The hemispherical photography showed very high values of *R*^2^, even in presence of the outlying value (*R*^2^ = 0.94), although it showed a marked and quite constant overestimation error (RRMSE = 99.46%), suggesting the need to introduce a correction factor specific for vineyards. During the smart scouting, PocketLAI showed its reliability to monitor the spatial-temporal variability of vine vigor in cordon-trained systems, and showed a potential for a wide range of applications, also in combination with remote sensing.

## 1. Introduction

Leaf area index (LAI, total one-sided area of leaf tissue per unit ground surface; [1]) is a quantitative descriptor of canopy density and it is important index for monitoring vine (*Vitis vinifera* L.) vigor and for providing precious information to support vineyard management. Leaf area density is significantly correlated with radiation interception and light environment in the fruit zone [2], in turn affecting the canopy microclimate and the plant carbon balance, as well as yield and grape composition [3,4]. For these reasons, attempts have been made to forecast vine yields using remotely sensed vegetation indices (i.e., normalized difference vegetation index, NDVI) related with plant vigor and LAI [5]. Concerning management support, the relationship between LAI and crop coefficient (Kc) was used to quantify water requirements and define irrigation strategies [6]. Moreover, recent studies have used the relationship between canopy density and disease incidence [7] to adjust pesticide dose [8]. In this context, Walklate et al. [9] developed a system for supporting the efficient use of pesticides in vineyards based on descriptors of canopy density, such as plant LAI, row and vine spacing. Experimental evidences pointed out that the practice, commonly adopted in viticulture, to apply a constant dose of pesticide per unit area leads to foliar deposits that consistently decrease when vine LAI increases [9,10,11,12]. Therefore, the application of a unique pesticide dose can results in over- or under- plant protection, with the negative economic and environmental impacts. In this context, Siegfried et al. [13] highlighted the importance of adapting the dosage following a LAI-based approach.

However, the LAI determination in vineyards using indirect methods (suitable in operational contexts) is constrained by the peculiar canopy structure, that is mostly vertically trained with a marked row structure (i.e., the cordon-trained system, where the cordon is the vines woody framework arising from the top of the trunk, trained along horizontal wires, and on which arms are borne).

The indirect methods are based on the measurement of light transmission through the canopy and, in case of vineyards, they can be affected by the discontinuity of the canopy and by the presence of leaves clumped in walls. These aspects violate the main assumption behind the light transmittance models used within indirect methods [14], also known as turbid medium assumption [15]. Johnson and Pierce [16] reported systematic under-estimation of vineyard LAI using LAI-2000 (PCA; LI-COR Inc., Lincoln, NE, USA). Lopez-Lozano and Casterad [17] assessed several measurement protocols for the SunScan ceptometer (Delta-T Devices, Cambridge, UK) in vineyards, underlining the need of introducing information on canopy structure for reliable measurements and the strong impact of sun position on the uncertainty in LAI estimates. Similarly, remote sensing techniques demonstrated to be partly unsuitable to derive vine LAI estimates, although to a different extent for the different remote sensing platforms. In general, the large spatial heterogeneity of a row-structured canopy and variability in the background (e.g., more or less vegetated, bare soil) markedly affects spectral reflectance, leading to unstable relationships between vegetation indices, such as NDVI and LAI [18,19,20]. Therefore, vegetation indices need to be related with ground-based LAI measurements to derive spatially distributed LAI maps, the latter being useful, e.g., to define the dosage of plant protection products [21,22,23,24].

For the reasons above, the monitoring of vines vigor for operational purposes is mainly carried out by estimating leaf area with simplified techniques characterized by low accuracy, such as those based on canopy leaf layer count [4] and on allometric relationships with shoot number and length [20].

An inexpensive alternative, to a certain extent unexplored for grapevine, could be represented by the digital hemispherical photography (DHP) technique [25]. DHP is based on permanent image acquisition through a fish-eye lens and on the use of specific software for image processing [26]. However, most of these software packages are based on the interactive (manual) application of a visually-selected threshold for the whole image, introducing a potential source of inconsistency due to the user’s subjectivity; moreover, they are not resource-efficient when a large number of images needs to be processed [27]. Therefore, DHP is not able to provide data in real-time and the semi-automatic segmentation of images can be affected by user’s skills and subjectivity.

An alternative inexpensive solution to get real time LAI data in a fully automatic way for both research and operational purposes was proposed by Confalonieri et al. [28]. They developed a smartphone application called PocketLAI, based on the inversion of the Warren Wilson [29] light transmittance model and on the gap fraction estimates via unsupervised segmentation of image acquired below the canopy at 57.5°. The image acquisition at this particular directional configuration demonstrated—both theoretically and experimentally [30]—its independence from leaf angle distribution and its capability to minimize leaf clumping effect in case of row crops [30]. PocketLAI showed good accuracy for rice LAI estimates in comparative evaluations with LAI-2000 and AccuPAR ceptometer (Decagon, Pullman, WA, USA). In further studies, the smart app demonstrated its effectiveness also for herbaceous canopies markedly deviating from the ideal assumption (random distribution of infinitely small leaves) behind its simplified transmittance model [31], for a variety of broad-leaf tree canopies [32] and for seasonal monitoring purposes [33].

The availability of a smart-app—characterized by low-cost, able to provide LAI estimates in absolute terms, in real-time, without requiring time consuming post-processing or the definition of parameters/settings—could represent an useful and user-friendly tool for operational purposes in the viticulture sector, suitable for the agricultural stakeholders (e.g., producers, insurance agents, etc.).

Recently, another smart-app (VitiCanopy) [34] was developed to estimate vine canopy vigor and porosity. VitiCanopy is based on the acquisition of images from below the canopy similarly to DHP, and on their automatic processing, analogously to PocketLAI. However, the VitiCanopy user needs to specify the gap fraction threshold, meaning the percentage of image pixel that corresponds to sky, before taking the measurements. VitiCanopy showed good agreement with LAI-2000 [34], although the authors underlined the influence of the cordon included in the image on the app accuracy. The resulting overestimation depends on intrinsic factors concerning the cordon size (e.g., vine age and training system) and on aspects related to the measurement protocol (i.e., the distance between the camera and the cordon). The authors highlighted that the assessment of the contribution of the cordon is not necessary when the aim is to monitor relative differences of vigour in time and space. On the other hand, when absolute LAI values are required, the authors suggested the calibration of a case-specific correction factor using measurements carried out on bare cordons during dormancy to quantify the cordon contribution. In this respect, the automatic acquisition of images at 57.5° implemented in PocketLAI should make the latter free from any need of parameterization or calibration, since images are captured pointing the camera toward the leaves wall and thus excluding the cordon.

The aims of this study were to assess the reliability of vineyards LAI values retrieved with PocketLAI and to develop a specific measurement protocol for grapevine. The performances of PocketLAI were assessed against destructive measurements and compared with those of DHP. Moreover, the possibility of using in-situ PocketLAI measurements and Normalized Difference Vegetation Index (NDVI) maps for obtaining spatially distributed LAI maps was also explored via smart scouting.

## 2. Materials and Methods

### 2.1. Survey Scheme

The study was carried out during 2015, in the rainfed experimental vineyard of Riccagioia s.c.p.a. (Pavia Province, Italy; Lat. 44.98 N, Long. 9.09 E, 138 m a.s.l.). Vines were planted in 2008 with a row and vine spacing respectively of 2.4 m and 1.0 m, cultivated using the Guyot system and unilateral cordon-pruning. The vineyard covers an area of about 1 ha and it is characterized by a pronounced heterogeneity, mainly due to the use of different cultivars (*Vitis vinifera*: cv. Barbera and Chardonnay) and rootstocks (hybrids of *Vitis berlandieri* and *Vitis riparia*) within each row, as well as to different vine care practices (with or without green pruning).

Sampling areas of different vigor were identified during six surveys carried out from the emergence of inflorescence to the beginning of ripening, in order to monitor the evolution of vine canopy during the crop cycle and provide a wide range of LAI values on which testing the indirect methods against the destructive measurements. In particular, the surveys were performed at the following growth stages of the BBCH phenological scale: 56, 57, 61, 74, 77, 81 [35].

During each of the first five surveys, three sampling areas of different vigor were identified, and three vines were sampled for each area. The sampling areas were characterized by cv. Barbera vines, all subjected to green pruning. The differences of canopy vigor in-space were mainly due to the use of three different rootstock hybrids (Table 1), and in-time to the different surveyed growth stages.

During the sixth survey, five sampling areas of different vigor were identified on the basis of the relative differences of canopy vigor described by a high resolution NDVI map, and—in each of these areas—two vines were sampled. The NDVI map was derived using a sensor placed on a four-wheeler quad-bike that detected the spectral response of the vines while moving through the rows. This last survey had the aim to extend the range of LAI values on which test the performance of indirect methods and to explore the possibility to combine PocketLAI with remote sensing data. In this case, the spatial variability of canopy vigor was due to the presence of different combination of cultivar × rootstocks (Table 1), and to the involvement of vineyard areas not subjected to green pruning.

On the sampled vines, PocketLAI and DHP estimates were first taken, followed by the collection of destructive samples obtained by stripping all leaves from the canopies. Moreover, during the first five surveys, the number of shoots per plant was determined, and the average number of leaves per shoot was estimated by counting the leaves on a sample of five shoots. These two measures were summarized in a descriptive index (theoretical leaf number; TL) given by the shoot number multiplied by the average number of leaves per shoot.

### 2.2. Indirect LAI Estimates

The method implemented in PocketLAI and the app functioning were described in previous papers [28,31,32] (documentation available at the http://www.cassandralab.com/mobiles/1 and licensing at info@cassandralab.com). Using the accelerometer and the device camera, PocketLAI automatically takes images from below the canopy at a view angle of 57.5° while the user is rotating the device along its main axis. The gap fraction is derived using a fully automatic segmentation algorithm expressly developed to detect the sky pixels according to their chromatic values in a Hue- Saturation-Brightness (HSB) color space. The LAI value was retrieved according to the light transmittance model proposed by Warren-Wilson [29] and recently discussed by Baret et al. [30].

**Figure 1:**
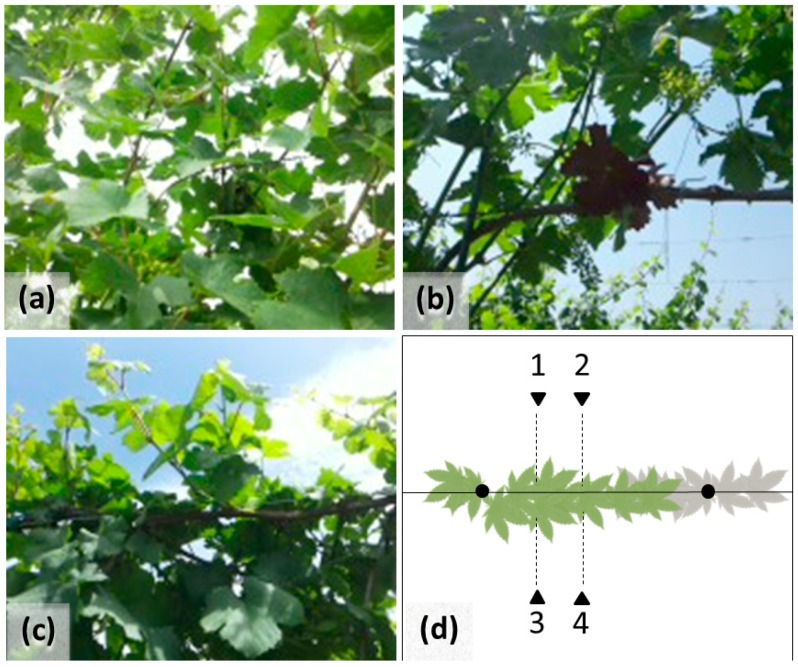
Example of correct image captured with PocketLAI following the protocol (**a**); and wrong images that include the space below (**b**) or above (**c**) the vertical trained canopy of Vitis vinifera. Protocols for LAI data acquisition (**d**): dark triangles and dotted lines = device orientation; black points vineyard poles; continuous line = vineyard row; green leaves = measured vine; grey leaves = adjacent vine.

For this study, PocketLAI was installed on an inexpensive Samsung GT-i9105 Galaxy S II Plus smatphone. In the specific case of vineyard, measurements were taken by positioning the device at about 15 cm below the canopy and at a distance of 0.4 m from the row, orienting the device toward the vines (Figure 1a). This allowed excluding the empty space between the bottom of the canopy and the soil surface (Figure 1b), as well as the sky above the canopy (Figure 1c). For each plant, four measurement replicates were acquired by processing two shots per row side while moving along the row at about 20 and 40 cm from the vineyard pole (Figure 1d). Averaging the four replicates, the LAI of each vine was obtained. Then, averaging the LAI of the sampled vines, the mean plant LAI (LAIp) in each sampling area was calculated.

The LAIp provided by PocketLAI was converted in vineyard LAI (LAIv) using Equation (1):

(1) $$\mathrm{LAIv}=\mathrm{LAIp}\frac{2D}{R}$$

where D (m) is the horizontal distance of the device from the cordon, and R (m) is the row-spacing.

The equation was derived from the assumption that the LAIp obtained from PocketLAI is given by the ratio between the plant leaf area and the unit ground surface considered by the smart-app (Equation (2)). The latter is the vine-spacing multiplied by the inter-row width considered by the PocketLAI on both sides of the row, corresponding to the double of the distance between device and row (0.8 m).

The value of leaf area per plant assessed by PocketLAI was retrieved (Equation (3)) and referred to the unit of ground surface provided for single vine in the vineyard, dividing by vine-spacing × row-spacing [16] (Equation (1)). The result is the conversion of LAIp in LAIv.

(2) $$\mathrm{LAIp}=\frac{\mathrm{LA}}{2D\times V}$$

(3) LA=LAIp×2D×V

where LA (m^2^) is the plant leaf area, V (m) is the vine-spacing, D (m) is the horizontal distance of the device from the cordon. The hemispherical images were collected through fisheye lens, using a protocol similar to that used for PocketLAI. The camera was positioned below the canopy along the row line, with the lens oriented upward, acquiring two images from each plant, moving horizontally at 20 and 40 cm from the pole. The set of fisheye images from each sampling area was processed using the CAN-EYE software (v 6.314; http:www.avignon.inra.fr/can_eye) [15], since it demonstrated to be one of the best packages in a comparative study performed by Liu et al. [36]. The segmentation of the images targeted the discrimination of green organs from the sky background and from the brown pixels, in order to exclude from the LAI estimate the contribution of the cordon and other minor woody components. The ‘true LAI’ values provided by the software were used as LAIv. The ‘true LAI’ is defined as the LAI value assessed by CAN-EYE taking into account the computation of average leaf inclination angle and clumping parameter.

### 2.3. Destructive LAI Measurement

The LAI estimates from destructive sampling followed the approach suggested by Breda et al. [37] and Johnson and Pierce [16]. Destructive LAI measures were collected immediately upon completion of indirect estimates by removing all the leaves at the petiole from each sample plant. Then, for each plant, the leaf fresh weight (W) was determined and a disc of known area (0.01 m^2^) was obtained for each of the leaves in a 40-leaf subsample including leaves of different ages. The 40 discs per plant were weighed and the specific leaf area (SLA; m^2^·g^−1^ f.w.) was computed. The obtained values of SLA, W and plant density were used to derive the vineyard LAI (LAIv) according to Equation (4):

(4) $$\mathrm{LAIv}=\frac{W\cdot \mathrm{SLA}}{R\cdot S}$$

Therefore, similarly to Equation (1), in order to compute LAIv, the leaf area per plant was referred to the unit of ground surface provided for single vine in the vineyard [16].

### 2.4. Data Analysis

For each sampling area, the agreement between mean LAIv values determined using destructive and indirect methods was quantified using the following metrics: relative root mean square error (RRMSE; 0 to +∞, optimum 0) [38], mean absolute error (MAE; 0 to +∞, optimum 0) [39], modelling efficiency (EF; −∞ to +1, optimum +1) [40], coefficient of residual mass (CRM; from −∞ to +∞ , optimum 0; if positive means underestimation and vice versa) [41]. Moreover, parameters of the linear regression equation between LAIv values estimated with direct and indirect methods were calculated, as well as between LAIv values and TL data. Moreover, for the sixth survey, a relationship between PocketLAI estimates and NDVI data was computed and used to derive spatially distributed LAI values for the entire vineyard.

## 3. Results

### 3.1. Performance of Indirect Methods

**Figure 2:**
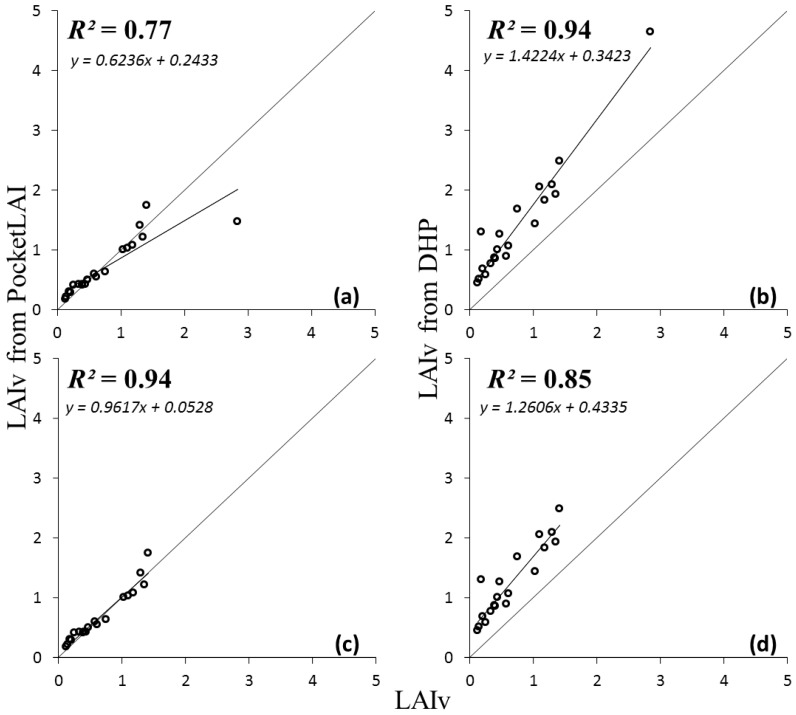
Agreement between LAIv data observed with direct measurement and those estimated with PocketLAI (**a**,**c**) and hemispherical photography (DHP) (**b**,**d**); considering the whole dataset (Dataset-1; **a**,**b**) and excluding the outlier value of a very high-vigour sampling area (Dataset-2; **c**,**d**).

The LAIv values obtained from destructive measurements during the campaign ranged from 0.13 to 2.84, in agreement with those normally reported in commercial vineyards [16,17,25]. The results of the comparison between LAIv values from PocketLAI, DHP and destructive measurements are shown in Table 2 and Figure 2.

Considering the whole dataset of surveys (Table 2, Dataset-1; Figure 2a), PocketLAI values were in agreement with those from destructive measurements, with a highly significant correlation (*p* < 0.001) (*R*^2^ = 0.77) and practically without under- or over-estimating behaviors (CRM = 0.06). However, the comparison was largely affected (RRMSE = 43%) by a single value (LAIv = 2.84), corresponding to the sampling area characterized by the highest vigor, identified via smart scouting before the last survey using the NDVI map. The highest LAI value corresponds to the sampled area of the vineyard untreated by the green pruning, and therefore it can be considered an outlying data.

The exclusion of this single value from the dataset led to greatly improve the coherence between PocketLAI and destructive LAIv values (Table 2, Dataset-2; Figure 2c). The new values of the agreement metrics decidedly approximated their optimum values: *R*^2^ was 0.94, RRMSE reached 17%, and EF was 0.93.

Considering the whole dataset (Table 2, Dataset-1; Figure 2b), DHP showed a systematic over-estimation (CRM = −0.87), with high RRMSE (99.46%) and negative value of EF (meaning that the average of destructive measurements is a better estimator of each LAI value compared to the DHP values). On the other hand, DHP showed high correlation with destructive LAIv values (*R*^2^ = 0.94) and its performances were not affected by the outlier value (i.e., no improvements detected repeating the analysis with the exclusion of the outlying value; Table 2, Dataset-2; Figure 2d).

Both indirect methods showed highly significant correlations between LAIv estimates and the descriptor of plant vigour represented by TL (Table 2), with values of *R*^2^ similar to that achieved using the destructive method. However, a slightly better agreement between TL and LAIv was obtained for PocketLAI (*R*^2^ = 0.96, it was 0.85 for DHP).

**Figure 3:**
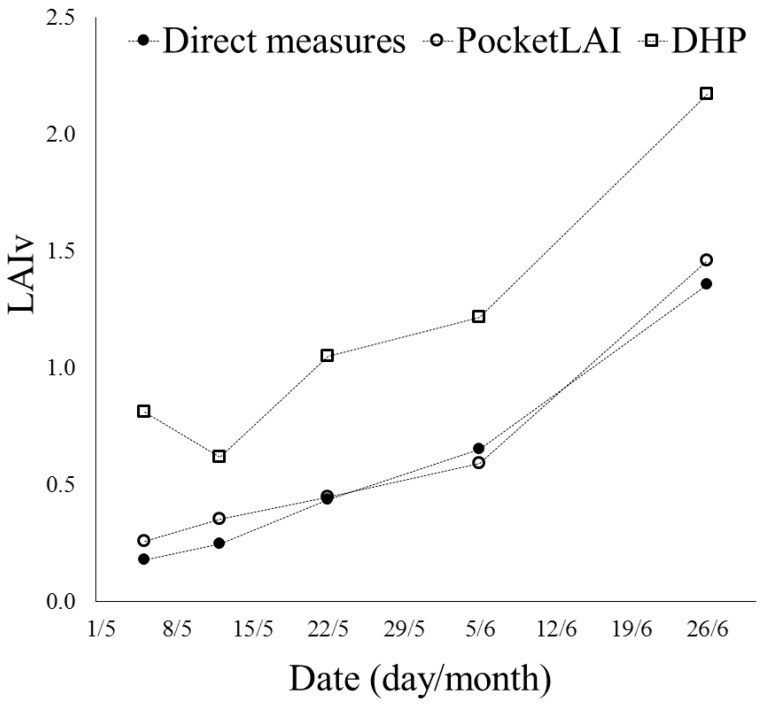
Average trend of LAIv observed during the monitoring of three rows from the beginning of May to the end of June, with direct and indirect methods.

Figure 3 shows the increasing trend of the mean LAIv during the growing season, from the beginning of May to the end of June (i.e., during the first five surveys). Data confirmed what discussed above, with the constant over-estimation of LAIv provided by the DHP, whereas PocketLAI estimates were always very close with destructive measurements. Moreover, at the second survey, the DHP detected an average LAI decrease of 0.19 not confirmed by the other measurement methods. The inconsistency was due to the sky lighting during the acquisition of hemispherical images. During the second survey, the conditions (sunny day and survey carried out with sun at the zenith) led to high level of sunlight affecting the evenness of foliage lighting, with resulting uncertainty in image segmentation. This led to underestimation errors, as already observed by other authors [26,42]. The PocketLAI view angle of 57.5° minimized the effect of those conditions because, contrarily to hemispherical photography, the device camera was not oriented directly toward the zenith during image acquisition.

### 3.2. PocketLAI and NDVI

**Figure 4:**
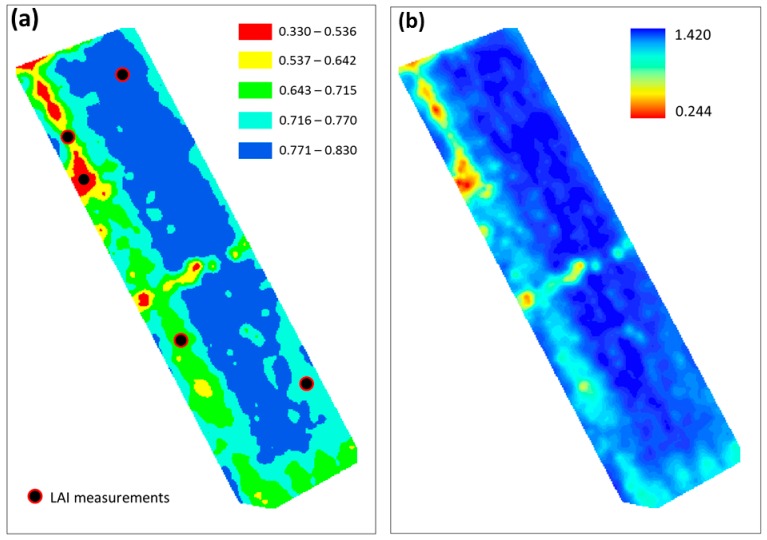
NDVI map of the vineyard (**a**), with pixels clustered in five classes as shown by the legend and indications on where PocketLAI estimates were collected, and the corresponding LAIv map (**b**) derived from the relationship between NDVI and PocketLAI measurements in the five points representative of each NDVI class.

The pixels of the georeferenced NDVI map of the vineyard were clustered into five classes using the ArcGIS v.10 package, and PocketLAI estimates were collected at the sixth survey in sampling area representative of each class (Figure 4a). A regression equation (*R*^2^ = 0.90) was then derived between the LAIv obtained from PocketLAI and NDVI values in the sampling areas (indicated by black circles in Figure 4a). The regression equation was then used to convert all the other NDVI pixels of the map into LAIv values, using the spatial analysis tool of ArcGIS for raster reclassification to obtain spatially distributed LAI data (Figure 4b). Figure 4b shows the resulting high resolution vineyard LAI map, derived from the combination of the vegetation index and only five PocketLAI estimates (smart scouting).

## 4. Discussion

In general, the LAI values estimated using PocketLAI and those measured using destructive method were consistent, with a coefficient of determination ranging from 0.77 (Figure 2a) to 0.94 (Figure 2d), depending on the inclusion or not in the analysis of a vineyard LAI close to 3 (LAIv = 2.84 with destructive method), observed in a unusually high-vigor sampling area due to the absence of green pruning applications. This value, converted in vine LAI (Equation (2)), corresponds to an observed plant LAI close to 7 (LAIp = 6.8).

This value can be considered as outlying in vineyards, as confirmed, e.g., by the LAI ranges reported by Pergher and Petris [12]. For similar vine training systems, this study shows LAI values ranging from 0 to 4, measured across many seasons (from 1993 to 2005) from leaf development (code 13 BBCH scale) to the beginning of ripening (code 81 BBCH scale).

Excluding this value, the maximum vineyard LAI observed was below 2 (LAIv = 1.41 with direct method), corresponding to a vine LAI lower than 4 (LAIp = 3.4). These results confirmed the tendency of PocketLAI (and of most indirect methods based on the inversion of light transmittance models) to underestimate high LAI values because of a saturation effect [26]. In case of PocketLAI, the effect was already discussed by Francone et al. [31] for dense maize and giant reed canopies. In that case, the authors obtained pronounced underestimations especially in case of giant reed canopies exceeding 5. The reasons were the saturation effect and the strong deviation of industrial giant reed plants from the ideal assumption behind light transmittance models (randomly distributed infinitely small leaves). The same explanation is valid for the case of vineyard (leaves clumped in walls). However, PocketLAI can be considered a suitable tool for monitoring *Vitis vinifera* LAI development, since it showed inaccuracies only in an uncommon case of vine untreated with green pruning. This practice is usually adopted in viticulture, in order to decrease the shading on the grapes and create microclimatic condition inside the canopy unfavorable to pest infection [43].

Concerning the smart scouting activities, the coupling between NDVI map and in-situ LAI data, measured with PocketLAI, proved to be an interesting approach able to provide a description of the vineyard canopy LAI in absolute terms. The possibility to convert an NDVI map, able to describe differences in space only in relative terms, to a LAI map acquires a special relevance when the optimization of farming practices requires a description of the vineyard based on absolute values, like in the case of pesticide dose adjustment.

In case of DHP, the correlation between indirect estimates and destructive measurements was fully satisfactory (*R*^2^ = 0.94) and the method did not show saturation effect, although estimates were consistently affected by a relevant overestimation error. The overestimation was quite constant and independent from absolute LAI values, since it was observed throughout the season. Similar results were found by Lopez-Lozano et al. [25], who compared different tools for LAI estimation in vineyards, including DHP. The authors discussed that the ‘true LAI’ values provided by the same software we used (CAN-EYE) led to overestimating destructive LAI values by a factor around 1.6 (the value we obtained here was around 2.6). These results suggested the possibility to apply a specific correction factor for DHP improving the performance of the method for the vineyard case.

Neither PocketLAI nor DHP showed changes in reliability for different phenological stages. Excluding the outlying value discussed above, differences in mean absolute error never exceeded 0.17 and 0.44 for PocketLAI and DHP, respectively, and no trends were observed for the evaluation metrics as a function of development stage.

## 5. Conclusions

Both indirect methods based on digital image processing, i.e., the PocketLAI smart-app and hemispherical photography, proved their reliability for monitoring the spatial and temporal variability of vine vigor in cordon-trained system and to provide LAI values. However, DHP highlighted the need to assess a correction factor because of overestimation errors already discussed for vineyards [25], whereas PocketLAI showed a certain saturation effect for vine LAI approaching or exceeding 5 or 6. However, the latter is an uncommon condition with respect to the most widespread vine training systems, because the green pruning lead to lower LAI values.

Moreover, with respect to DHP, PocketLAI entails the advantages of automatic (completely unsupervised) and real-time provision of LAI data and the suitability for operational purposes, in addition to its low-cost and user-friendliness. In this context, PocketLAI shows a potential for a wide range of applications, such as the spatial-temporal optimization of the agronomic inputs, especially in combination with remote sensing products.

## Figures and Tables

**Table 1:** Survey scheme.

Date	BBCH-Stage	Row Genotype	Number of Sampled Vines
5 May	56	cv. Barbera (rootstocks: AT84 × Kober 5bb)	3
		cv. Barbera (rootstocks: AT84 × SO4)	3
		cv. Barbera (rootstocks: AT84 × 420A)	3
12 May	57	cv. Barbera (rootstocks: AT84 × Kober 5bb)	3
		cv. Barbera (rootstocks: AT84 × SO4)	3
		cv. Barbera (rootstocks: AT84 × 420A)	3
22 May	61	cv. Barbera (rootstocks: AT84 × Kober 5bb)	3
		cv. Barbera (rootstocks: AT84 × SO4)	3
		cv. Barbera (rootstocks: AT84 × 420A)	3
5 June	74	cv. Barbera (rootstocks: AT84 × Kober 5bb)	3
		cv. Barbera (rootstocks: AT84 × SO4)	3
		cv. Barbera (rootstocks: AT84 × 420A)	3
23 June	77	cv. Barbera (rootstocks: AT84 × Kober 5bb)	3
		cv. Barbera (rootstocks: AT84 × SO4)	3
		cv. Barbera (rootstocks: AT84 × 420A)	3
20 July	81	cv. Chardonnay (rootstocks: R8 × M3)	2
		cv. Chardonnay (rootstocks: R8 × M2)	2
		cv. Chardonnay (rootstocks: AT84 × SO4)	2
		cv. Barbera (rootstocks: AT84 × Kober 5bb)	2
		cv. Barbera (rootstocks: AT84 × SO4)	2

**Table 2:** Agreement between LAIv estimated with PocketLAI, hemispherical photography (DHP), and direct measurements, considering the whole dataset (Dataset-1) and excluding the outlying value of a very high-vigor sampling area (Dataset-2), and agreement between LAIv measured with different methods and the theoretical number of leaves (TL). Legend: MAE: mean absolute error; RRMSE: relative root mean square error; EF: modelling efficiency; CRM: coefficient of residual mass.

	Agreement between LAI Measurement Methods					
	PocketLAI vs. Direct Measures		DHP vs. Direct Measures		PocketLAI vs. DHP	
	Dataset-1	Dataset-2	Dataset-1	Dataset-2	Dataset-1	Dataset-2
*R*^2^	0.77 *	0.94 *	0.94 *	0.85 *	0.68 *	0.84 *
MAE	0.15	0.09	0.66	0.60	0.57	0.57
RRMSE	43.00	17.27	99.46	100.79	50.39	50.39
EF	0.74	0.93	−0.40	−1.29	−0.14	−0.14
CRM	0.06	−0.04	−0.87	−0.93	0.46	0.46
	**Agreement between plant vigour descriptors**					
	**PocketLAI vs. TL**		**DHP vs. TL**		**Direct measures vs. TL**	
*R*^2^	0.96 *		0.85 *		0.92 *	

* *p* < 0.001.
